# Estimating the cost-effectiveness of detecting cases of chronic hepatitis C infection on reception into prison

**DOI:** 10.1186/1471-2458-6-170

**Published:** 2006-06-27

**Authors:** Andrew J Sutton, W John Edmunds, O Noel Gill

**Affiliations:** 1 Health Protection Agency, Centre for Infections, 61 Colindale Ave, London NW9 5EQ, UK; 2 Department of Biological Sciences, University of Warwick, Coventry CV4 7AL, UK

## Abstract

**Background:**

In England and Wales where less than 1% of the population are Injecting drug users (IDUs), 97% of HCV reports are attributed to injecting drug use. As over 60% of the IDU population will have been imprisoned by the age of 30 years, prison may provide a good location in which to offer HCV screening and treatment. The aim of this work is to examine the cost effectiveness of a number of alternative HCV case-finding strategies on prison reception

**Methods:**

A decision analysis model embedded in a model of the flow of IDUs through prison was used to estimate the cost effectiveness of a number of alternative case-finding strategies. The model estimates the average cost of identifying a new case of HCV from the perspective of the health care provider and how these estimates may evolve over time.

**Results:**

The results suggest that administering verbal screening for a past positive HCV test and for ever having engaged in illicit drug use prior to the administering of ELISA and PCR tests can have a significant impact on the cost effectiveness of HCV case-finding strategies on prison reception; the discounted cost in 2017 being £2,102 per new HCV case detected compared to £3,107 when no verbal screening is employed.

**Conclusion:**

The work here demonstrates the importance of targeting those individuals that have ever engaged in illicit drug use for HCV testing in prisons, these individuals can then be targeted for future intervention measures such as treatment or monitored to prevent future transmission.

## Background

Hepatitis C (HCV) is a blood-borne viral infection that affects the liver the causative agent of which was identified only in 1989[1]. The virus is spread when blood from an infected person gets into the bloodstream of another. In the UK blood donations have been screened for HCV since September 1991. Consequently, it is now very difficult to acquire HCV infection by blood transfusion. Unlike many other blood borne viruses, sexual transmission is thought to be relatively rare[2]. HCV is an important problem in current and previous injecting drug users (IDU)s who are exposed to the virus through the sharing of needles and injecting paraphernalia[3]. It has been shown that the prevalence of HCV infection in current and former IDUs in England and Wales is approximately 41%[4]. Co-infection of blood-borne viruses is also a problem for IDUs, it has been found for IDUs in England and Wales in 2000–2003 that approximately 30% of current IDUs were co-infected with HBV and HCV, and approximately 1% were co-infected with HCV and HIV[4].

In the EU, data from 63 data sources obtained covering the period 1996–2002 in 14 countries of the EU were available (no data was available from Sweden)[5]. The HCV prevalence in IDUs was found to be as low as 35.7% in the UK and as high as 80.6% in Denmark with the mean across 14 European Union countries being 65.1%. While an alternative study[6] that reviewed literature from studies in the European Union from 1990–2000 found that the incidence rates for HCV in the IDU population ranged from 6.2 to 39.3 per 100 person years.

Using data on the number of deaths due to opiate overdose it has been estimated that the prevalence of opiate users/injecting drug users has increased from less than 20,000 in 1980 to between 100,000 and 150,000 in 2000 in England and Wales[7]. Alternative estimates of the prevalence of problematic drug use in the UK undertaken in 2001 have ranged from 161,000–266,000[8].

Due to the illegality of injecting drug use and the close association between crime and drug manufacture, trafficking, supply, and use, those involved with injecting illicit drugs frequently pass through the penal system[9]. This suggests that prison is a good location to administer intervention measures targeting the IDU population.

It was found in a previous unlinked anonymous surveillance survey[4] that 49% of IDUs self reported that they were unaware of their HCV infection while an alternative study[10] found that in IDUs that tested positive for HCV only 23% of them were aware of their positive status. This lack of awareness of their infection status imposes a considerable barrier to treatment and transmission prevention.

There are a variety of alternative intervention measures that target illicit drug use[11]; Prevention programmes aim at the reduction of initiation or progress from experimental to regular drug use, treatment programmes aim to reduce drug use in experienced users, enforcement programmes aim at reducing the supply of drugs by targeting traffickers or dealers for arrest and reducing the demand for drugs by targeting the buyers of drugs, while harm reduction programmes seek explicitly to reduce the adverse consequences of drug use.

The work here considers the cost effectiveness of a range of alternative HCV case-finding scenarios implemented on reception into prisons in England and Wales to identify persons infected by chronic HCV (HCV RNA positive). Each scenario will be compared by considering the cumulative cost of identifying a new case of HCV (HCV RNA positive) and how this cost changes over time as previously tested individuals return to prison. Incremental cost-effectiveness analysis will also be undertaken.

## Methods

### Model structure

The model here is adapted from the model of the HBV vaccination programme in prisons. This model describes the flow of individuals through prison including the risk of incarceration for IDUs and non-IDUs and is stratified by injecting status and age[12]. The current IDU population described here has been subdivided into new initiates to injecting and experienced IDUs. New initiates to injecting are defined as those IDUs with an injecting career length of less than a year, and experienced IDUs are those individuals with an injecting career length of greater than one year. The force of infection estimates; defined as the per capita rate that susceptibles acquire infection, for both new initiates and experienced IDUs are taken from a previous study[13]. It is assumed that the force of infection rates are constant over time and independent of prison status. While it is acknowledged that the risk of blood-borne virus infection amongst IDUs inside prison may be significantly higher than in the community[14], obtaining reliable force of infection estimates that distinguish between prison status have not been obtained. Due to the small proportion of HCV infections that have an identified risk factor other than injecting drug use in England and Wales[15], it is anticipated that the force of infection in non-IDUs will be extremely low, and so for this reason it is assumed that only IDUs can become infected by HCV with the incidence of new infections in the general population (non-IDUs) assumed to be zero. For those persons that are infected by HCV it is assumed at the time of infection that 80%[2] become HCV RNA positive, although this assumption will be tested during sensitivity analysis.

### HCV case-finding coverage

Currently there is no significant ongoing HCV case-finding on reception into prisons across England and Wales. It is assumed therefore that HCV case-finding coverage on prison reception is expanded over time to reflect the rolling out of an HCV case-finding programme across prisons in England and Wales (1). shows the percentage of prison receptions in England and Wales where case-finding is undertaken over time assumed in this analysis.

### Case-finding pathway on prison reception

The case-finding pathway through prison reception proposed here is taken directly from a previous study using publicly available information describing the implementation of screening and treatment of HCV in the Isle of Wight prison cluster[9] although treatment is excluded from the pathway considered here. The study of the Isle of Wight Prison Cluster considers prisoners as they first attend a one hour health awareness lecture during which they are alerted to the risk factors for blood-borne viruses and are then invited to the healthwatch clinic which was set up in 1997 to provide counselling on blood-borne viruses for all new receptions to the Isle of Wight prisons. Testing and counselling undertaken in the healthwatch clinic on reception as described in the previous study[9] are applied here and described below (Figure 2 and Figure 3). In addition verbal tests are introduced along the screening pathway to investigate whether alternative HCV case-finding strategies may be more cost effective than offering testing to all individuals. Prisoners are given verbal tests regarding their previous injecting behaviour and results of previous HCV testing, with the answers to these questions used to judge whether the prisoners are eligible to receive serological HCV tests to establish their HCV status.

**Figure 1:**
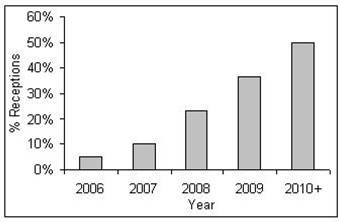
Assumed proportion of prison receptions covered by HCV case-finding.

**Figure 2:**
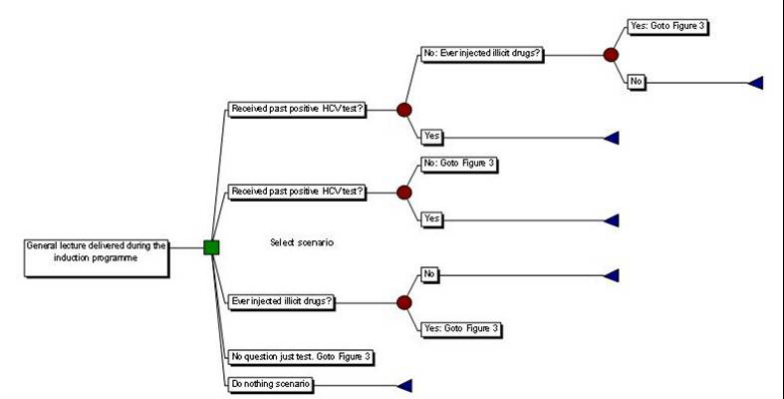
Schematic diagram of the case-finding scenarios used in this analysis.

**Figure 3:**
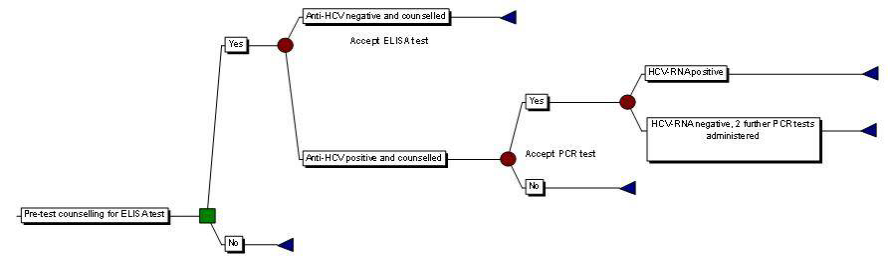
Pathway describing the administering of hepatitis C serological tests.

The case-finding pathway is described in (Figure 2 and Figure 3) and applies to all prisoners that are amongst the proportion on reception that are covered by the HCV case-finding programme (Figure 1). Initially all prisoners on reception into prison attend a one hour health awareness lecture alerting them to the risk factors of blood borne viruses. During each lecture it is assumed that 10 prisoners are present. Following this, prisoners are submitted to verbal tests to determine their eligibility to receive antibody tests. The verbal tests are a combination of the following, and represent the alternative case-finding scenarios considered here:

1. Have you received a positive HCV test previously?

2. Have you ever injected illicit drugs?

The first question is asked to establish whether the prisoner has been previously diagnosed with HCV. This question specifically asks about a previous positive test rather than simply a previous test, as a previous negative test is of no interest as it is likely that the prisoner may have been exposed to HCV in the mean time. The 2^nd ^question is used to establish whether the person has ever injected illicit drugs. Where verbal questioning is administered (scenarios one to three, see below), the time taken to question the prisoners is assumed to be independent of the number of questions asked and is assumed to take 5 minutes, although this will be examined during sensitivity analysis. Previous studies[10,16,17] have considered the sensitivity and specificity of IDUs responses to questions related to HCV positivity and the self-reporting of their illicit behaviours. The values taken at baseline and applied during sensitivity analysis are described in Table 2.

Following the verbal tests those prisoners that have been identified as being eligible to receive serological testing are assumed to be offered pre-test counselling. For those prisoners that are willing to accept serological testing an enzyme linked immunosorbant assay (ELISA) antibody test is assumed to be administered. Prisoners identified as having a positive antibody test are assumed to be informed that they have evidence of contact with HCV and are then offered a polymerase chain amplification (PCR) test for the presence of HCV viral RNA as a marker of ongoing infection. Those who are positive for HCV by antibody testing but negative on a single PCR test are assumed to be offered two further PCR tests as was adopted in the Isle of Wight Study[9], the costs of which are incorporated in the analysis.

Post-test counselling is administered to all prisoners on receipt of the results from the ELISA and PCR tests. Those with positive tests are assumed to be counselled on harm reduction and harm minimisation. Assumed time and staff allocations for each task on the case-finding pathway are shown in Table 2, where no reference was available to inform these values, reasonable assumptions were made with the impact of these being examined during sensitivity analysis.

### Community HCV testing and diagnosis

As a result of injecting treatment and prevention services in the community, it is possible for some HCV positive IDUs to become aware of their positive status as a result of testing in the community. Further to this, HCV positive individuals that develop symptoms associated with their infection may also have their HCV infection diagnosed in the community.

Data was considered from the Unlinked Anonymous survey 2003–2004 of IDUs (who injected in the previous four weeks prior to the survey) reporting whether they had ever received a blood test for HCV by career length. Assuming that this data is representative of the IDU population in the community and that no HCV testing had been undertaken in prison, a model was fitted to the data using maximum likelihood (fit not shown). The rate that IDUs in the community are tested and diagnosed for HCV in the community was estimated to be 0.15/IDU/year.

### Case-finding scenarios

Varying the verbal questions asked on prison reception allows us to compare six alternative case-finding scenarios used to identify individuals that are eligible to receive an offer of serological testing for HCV (the ELISA and PCR tests offered being the same for scenarios one-four, see above). A negative answer to any of the questions asked indicates that the person is ineligible for HCV serological testing. The verbal screening that distinguishes each scenario is described in Table 1:

Comparisons are made between each case-finding scenario by considering the cumulative cost per chronic HCV case (RNA positive) detected and how this varies over time. The complete parameter set describing the case-finding scenarios at baseline are shown in Table 2. The implication of these parameter selections on the model results are examined during sensitivity analysis with the values taken during sensitivity analysis also shown in Table 2. All costs are presented in year 2004 with discounting rates for both costs and benefits taken at 3.5% as recommended by the HM Treasury[18] although the impact of these are varied during sensitivity analysis (Table 2). The analysis here is considered from the perspective of the health care provider.

## Results

**Figure 4:**
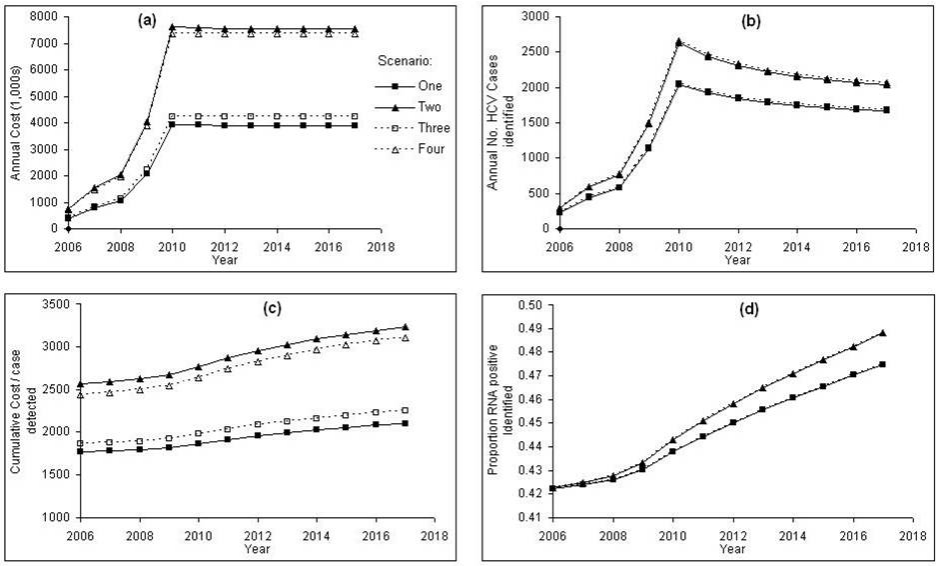
Taking parameter values as baseline values (Table 2), the results for the first four case-finding scenarios compared with the current do nothing (no HCV case-finding in prisons) strategy: a) the undiscounted annual cost of implementing each case-finding scenario, b) the undiscounted annual number of new HCV cases identified when implementing each case-finding scenario, c) the cumulative average cost per new HCV case detected with discounting, d) the proportion of those RNA positive that are identified over time.

It can be seen (Figure 4. c) that scenario one: identifying those individuals that have not received a past positive HCV test, and have ever injected illicit drugs is the most cost effective scenario over time. This shows the importance of identifying those individuals with a history of injecting drug use, which is the biggest risk factor for HCV infection. Least cost effective is scenario two: only identifying those individuals that have not received a past positive HCV test. For all scenarios the cost per new HCV case detected rises over time indicating that they become less cost effective as time passes. This is due to IDUs returning to prison that are already aware of their HCV infection, leaving a smaller proportion of individuals on reception into prison that have undiagnosed infection.

The undiscounted cost of implementing each case-finding scenario is shown in (Figure 4a) . For each case-finding scenario the cost increases up to a plateau in 2010 and then is broadly constant from then on. The initial increase and then plateau can be explained by the assumed expanding coverage of HCV case-finding on prison reception over time up to a constant coverage from 2010 onwards. It can be seen that scenarios two and four are far more costly than the remaining scenarios, however neither of these scenarios screens prisoners on prison reception for any previous injecting drug use, and as a consequence many more ELISA and PCR tests are administered for scenarios two and four than are necessary. A slight reduction in the cost was observed in scenarios one and two from 2010 onwards, this is due to persons having been diagnosed with HCV infection returning to prison and then being verbally screened for a past positive HCV test, hence reducing the costs further along each of the case-finding pathways.

From Figure 4d it can be seen that scenarios two and four identify the greatest proportion of individuals that are RNA positive over time, however these scenarios are not very cost effective due to the high costs required to administer them.

Results from the incremental analysis are reported in Table 3, where the alternative strategies have been ranked according to their cumulative discounted cost in 2017 and the incremental cost-effectiveness ratios have been calculated. This highlights that scenario two is the most cost-effective option and has the smallest budget impact (least cost).

### Sensitivity analysis

**Figure 5:**
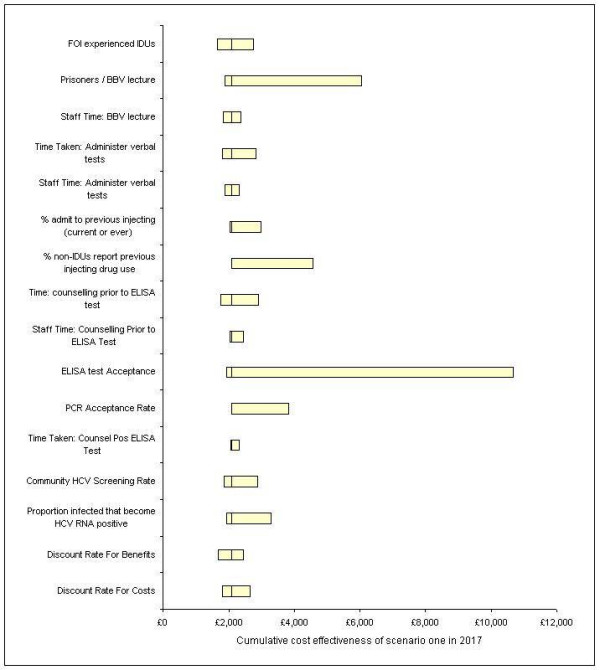
The impact on the cumulative average cost effectiveness of scenario one in 2017 compared to the current no case-finding strategy when applying one-way sensitivity analysis. The bars represent the costs when the upper and lower parameter estimates are implemented while the line through each is the baseline value.

Figure 5 shows the impact of one-way sensitivity analysis on the cumulative average cost per case detected in 2017 for case-finding scenario one compared with the current do nothing policy. In each case only those parameters that impact on the baseline value by greater than 10% are shown. It can be seen that in many cases the parameter values have little impact on the cost effectiveness of this scenario and in all cases this scenario was found to be the most cost-effective.

The parameters that have the largest impact on cost-effectiveness were found to be the number of prisoners that attend the blood-borne virus lecture on reception into prison, and the proportion of prisoners that accept an ELISA test. Also noted is the impact of non-IDUs (persons that have never injected drugs) reporting previous injecting drug use, however the authors could find no evidence of this occurring during previous screening programmes. It can be seen that the rate of HCV testing and diagnosis in the community and the force of infection estimates in the experienced IDUs also have an impact on the cost effectiveness estimates of scenario one. An increase in the rate of testing and diagnosis in the community and a reduction in the force of infection in experienced IDUs both result in the prison case-finding programme becoming less cost effective.

## Discussion

Using a Markov decision analysis model and a model of the flow of IDUs through prison the work here estimates the cost effectiveness of a number of alternative case-finding strategies including verbal screening for ever injecting drug use and for previous HCV testing. Results indicate that verbally screening for ever injecting illicit drugs and for ever having received a past positive HCV test was the most cost effective approach to establishing prisoners eligible for serological testing, while sensitivity analysis found that the proportion of eligible prisoners that accept ELISA testing had a significant impact on the cost-effectiveness of the case-finding scenarios.

It has been found that the case-finding scenarios described here all become less cost-effective over time, this being due to the impact of previously screened individuals returning to prison that do not need re-screening and the time taken to identify these prisoners. Although a reduction in annual costs were noted over time in some of the case-finding scenarios due to prisoners that had already received a positive serological test being identified on prison reception. For each scenario when the proportion of prison receptions covered by HCV case-finding had been assumed to have reached a constant the costs and benefits over time display only relatively small variation.

It is possible for an individual to become HCV RNA positive during a spell of imprisonment. This may occur for two reasons; either the individual was infected while in prison or the individual was infected outside prison but due to the natural history of HCV, HCV RNA was not detectable on reception into prison. HCV RNA can be detected within 1 to 2 weeks after exposure to the virus although HCV RNA positivity may appear much later at 30 to 40 days[19,20]. As two further PCR tests are administered to those individuals that are anti-HCV positive but negative for HCV RNA it is unlikely that individuals infected just prior to reception will remain undetected. If however it is felt that there is a significant risk of some prisoners becoming HCV RNA positive while in prison, then to allow these individuals to be offered treatment or counselling it may be necessary to administer some HCV testing during an individuals prison sentence or perhaps on discharge from prison.

The work has not given any consideration to the subject of treatment although it is acknowledged that the inclusion of the costs and benefits associated with treating individuals that have been identified with HCV in prison will play an important role in judging whether to implement an HCV case-finding programme on reception into prison. This study examines the best approach to identifying individuals that may be eligible for treatment and this can be taken forward when considering the implementation of treatment.

Results from sensitivity analysis showed the importance of encouraging eligible prisoners to accept the offer of an ELISA test, with a reduction in uptake having a large impact on cost-effectiveness. Intervention measures outside prison such as testing and diagnosis in the community or those that target IDUs aimed at reducing their at risk behaviour (and therefore the FOI) can have a negative impact on the cost-effectiveness of a prison based case-finding programme even though a prison based programme may well be more cost-effective than a programme based in the community. This shows the importance of coordinating intervention measures inside and outside prison to ensure the maximum effectiveness of both.

The work here has focused on identifying those individuals that may be at risk from HCV infection and then offering them an HCV test as appropriate, however it is possible that an individual may encounter further problems if they admit to injecting drug use, this in many instances may take the form of the social stigma associated with injecting drug use. It is hoped that admitting to previous injecting drug use will be seen in a positive light as individuals can then be targeted for drug related intervention measures or HCV treatment if necessary. To allow for the possibility that individuals may not give reliable answers to questions regarding injecting drug use or HCV status a range of values describing the sensitivity and specificity to individuals answers related to previous HCV testing and injecting drug use were considered. It was found that of importance to model results were the answers that individuals gave to the issue of previous injecting behaviour. However it is hoped that the one hour health awareness lecture described at the start of the screening pathway will provide a good opportunity in which to address individuals concerns regarding the revealing of potentially sensitive information.

A further account of screening for HCV in the prison population in England and Wales is described in a previous study considering the experience of screening in the Dartmoor prison[21]. In this study the authors describe data collected from a cohort of prisoners screened from 1 January 1998 to 30 June 2001 describing progress from test result to treatment. A key difference between the screening pathway described in Dartmoor compared to the Isle of Wight appears to be the additional two PCR tests administered at the Isle of Wight for those individuals that test anti-HCV positive but HCV RNA negative after a first PCR test. This means that the costs of the scenarios reported here may be less cost-effective than if the requirement to implement these two additional PCR tests was removed.

The approach to staff costs in this work has considered only the role of a doctor and nurse in implementing the alternative case-finding scenarios, while the estimates of the length of time taken to undertake the individual tasks along the case-finding pathways are inevitably subject to much variation. Considering only doctors and nurses is an obvious over simplification, with other members of staff such as prison chaplains and guards playing a role in the implementation of a prison based case-finding programme, However in mitigation the results from the sensitivity analysis have shown that staff costs and length of time required to accomplish each task play only a small part in variation in the estimates of the cost-effectiveness of the case-finding scenarios.

While treatment has not been considered here, there are still advantages in identifying those individuals that are anti-HCV positive. Any individual that is anti-HCV positive obviously has the potential to transmit infection particularly if the person is an IDU and continues to inject illicit drugs. An awareness of the infected status of these individuals is useful to services as they can then be targeted for intervention measures aimed at reducing the behaviour that leads to further HCV transmission. This may take the form of encouraging injecting cessation or the supplying of clean needles to reduce the risk of transmission due to the sharing of syringes. A further advantage of case-finding is that HCV positive individuals can be monitored so that they can be treated when treatment criteria have been met, which in the case of IDUs will be when the individual has ended their injecting career[22]. Finally the identification of HCV positive individuals on prison reception may also assist in identifying further positive individuals in the community through contact tracing exercises.

Future work should consider the cost-effectiveness of HCV treatment in a prison setting, including the possibility that some individuals may require a treatment pathway that involves continued treatment in the community following discharge from prison. The model here has not considered infection in the non-IDU population, and has assumed that all infection is as a result of injecting illicit drugs. While it is acknowledged that there is some HCV infection in the non-IDU population this is likely to be insignificant compared to injection related infection[4]. However it would be beneficial if FOI estimates could be obtained that consider the possibility of HCV infection due to risk factors not associated with injecting drug use, and the possibility of increased risk of infection in a prison setting. The model here could then be re-parameterised to reflect this new information.

## Conclusion

It has been shown here that verbally screening for ever injecting illicit drugs and for ever having received a past positive HCV test is the most cost effective approach to establishing prisoners eligible for HCV serological testing. The results from sensitivity analysis show the importance of encouraging eligible prisoners to accept ELISA testing with this having a significant impact on the cost-effectiveness of the case-finding scenarios.

## Competing interests

The author(s) declare that they have no competing interests.

## Authors' contributions

AJS contributed to designing and planning the study, created the cost-effectiveness model and carried out all analysis, interpreted the results and prepared the manuscript as the lead writer. WJE helped develop the model and interpret the data and results. ONG contributed to the study development and helped in the interpretation of the results. All authors read and provided comments on the manuscript and approved the final paper.

## Pre-publication history

The pre-publication history for this paper can be accessed here:



## Figures and Tables

**Table 1:** Summary of case-finding scenarios

**Scenario**	**Description**
One	Verbally screen for ever having received a past positive HCV test, and for ever having injected illicit drugs.
Two	Verbally screen for a past positive HCV test only.
Three	Verbally screening for ever injecting illicit drugs only
Four	No verbal screening
Five	No verbal screening and no testing (do nothing scenario)

**Table 2:** Model parameters and values used during sensitivity analysis

*Parameter*	*Baseline value*	*Sensitivity*	*Source (where available)*
λ_0 _= force of infection for new initiates	0.1608	0.1314–0.1942	[13]
λ_1 _= force of infection for experienced IDUs	0.0526	0.0310–0.0863	[13]
Cost of Doctor	£3.49/min (£209.40/hr)		GP:/home visit/min With Qualification costs With direct care staff costs[23]
Cost of Nurse	£54/hour		District Nurse (midpoint grade G)/hr with patient + extra costs[23]
			
*Time Taken for BBV lecture/prisoner*	10 patients/hr	1 patient/hr – 20 patients/hr	[9]
% Nurse time	50%	0–100%	
% Doctor time	50%	0–100%	
			
*Time taken to verbal test on reception into prison*	5 min	1–15 min	[24]
% Nurse time	50%	0–100%	[24]
% Doctor time	50%	0–100%	[24]
% Known HCV positive and say so	55% (midpoint)	23%–83%	23%[10], 83%[16],
% never received a positive HCV test but say they are HCV positive	1%	0%–10%	
% IDUs that report IDU use (current or ever)	75%	30–90%	Darke[17] reports that IDUs give reasonably reliable answers to questions about drug use.
% non-IDUs that report IDU use (current or ever)	0%	0–20%	
			
*Time Taken to Counsel prior to an ELISA test*	25 min	10–60 min	25 min[3] 10–60 min[24]
% Nurse time	50%	0–100%	[24]
% Doctor time	50%	0–100%	[24]
			
*Time taken to Administer ELISA test*	5 min	1–10 min	
% Nurse time	50%	0–100%	
% Doctor time	50%	0–100%	
Cost of ELISA test	£12	£5–20	Virus reference department, HPA, Colindale (2005)
% of those offered who accept ELISA testing	85%	10–100%	[24]
ELISA Sensitivity	97%	90–100%	[24]
ELISA Specificity	99%	90–100%	[24]
			
*Time taken to administer PCR test*	5 min	1–10 min	
% Nurse Time	50%	0–100%	
% Doctor Time	50%	0–100%	
% of those offered who accept PCR testing	100%	50%	Assuming that those that accept an ELISA test will then accept a PCR
PCR Sensitivity	100%	99%–100%	[24]
PCR specificity	100%	99–100%	[24]
Cost of PCR test	£57	£50–80	Virus reference department, HPA, Colindale (2005)
			
*Time taken to counsel the result of negative ELISA or PCR test*	5 min	5–15 min	
% Nurse time	50%	0–100%	
% Doctor time	50%	0–100%	
			
*Time taken to counsel the result of a positive ELISA test*	25 min	15–75 min	[24] 15–75 min[24]
% Nurse time	50%	0–100%	
% Doctor time	50%	0–100%	
			
*Time taken to counsel the result of a positive PCR test*	25 min	15–75 min	[24] 15–75 min[24]
% Nurse time	50%	0–100%	
% Doctor time	50%	0–100%	
			
Proportion infected with HCV that become HCV RNA positive	80%	54%–86%	[2,25,26]
Rate of HCV testing and diagnosis in the community	0.15	0.1–0.3	See text
Discount rate for costs	3.5%	0%–6%	HM Treasury[18]
Discount rate for benefits	3.5%	0%–6%	HM Treasury[18]

**Table 3:** Incremental cost effectiveness analysis of each case-finding scenario

*Case-finding Scenario*	*Cumulative discounted cost in 2017 (1,000s)*	*Cumulative discounted no. of cases of HCV identified in 2017*	*Incremental Cost Effectiveness*
Five (Do Nothing)	£0	0	
One	£28,192	13413	£2,102
Three	£30,444	13548	£16,625
Four	£53,123	17098	£6,388
Two	£54,670	16927	dominated
